# Metabolic challenges and key players in serpentinite-hosted microbial ecosystems

**DOI:** 10.3389/fmicb.2023.1197823

**Published:** 2023-07-24

**Authors:** Rabja Maria Popall, Anne Postec, Aurélien Lecoeuvre, Marianne Quéméneur, Gaël Erauso

**Affiliations:** Aix-Marseille Univ, Univ Toulon, CNRS, IRD, MIO, Marseille, France

**Keywords:** serpentinization, alkaline hydrothermal system, alkaliphile, hydrogenotroph, lithotroph, submarine alkaline vent theory, origin of life

## Abstract

Serpentinite-hosted systems are amongst the most challenging environments for life on Earth. Serpentinization, a geochemical alteration of exposed ultramafic rock, produces hydrothermal fluids enriched in abiotically derived hydrogen (H_2_), methane (CH_4_), and small organic molecules. The hyperalkaline pH of these fluids poses a great challenge for metabolic energy and nutrient acquisition, curbing the cellular membrane potential and limiting electron acceptor, carbon, and phosphorous availability. Nevertheless, serpentinization supports the growth of diverse microbial communities whose metabolic make-up might shed light on the beginning of life on Earth and potentially elsewhere. Here, we outline current hypotheses on metabolic energy production, carbon fixation, and nutrient acquisition in serpentinizing environments. A taxonomic survey is performed for each important metabolic function, highlighting potential key players such as H_2_ and CH_4_ cycling *Serpentinimonas*, *Hydrogenophaga*, *Methanobacteriales*, *Methanosarcinales*, and novel candidate phyla. Methodological biases of the available data and future approaches are discussed.

## 1. Introduction

The beginnings of life remain one of the most outstanding scientific issues and have been dubbed the “black hole at the heart of biology” (Lane, 2015). One of the central requirements for living systems is a continuous physicochemical disequilibrium driving biological activity (Russell et al., 1988). In marine alkaline hydrothermal systems, strong electrochemical gradients develop between the ultrabasic hydrothermal fluid rising from the deep subsurface, and the seawater. These gradients are maintained across the porous hydrothermal chimney wall, which can be compared to an osmotic membrane. It is hypothesized that this rudimentary proton motive force has driven chimney nanopores to develop into protocells at the emergence of life (Russell et al., 2010; Sojo et al., 2016). Modern ecosystems at hydrothermal vents might thus provide a glimpse into very early microbial life forms.

Most alkaline hydrothermal systems are formed in environments where mantle rocks have been tectonically uplifted and exposed, either above sea level or on the seafloor. Contact with water initiates serpentinization, a geochemical alteration of the ultramafic rock. This process yields large amounts of hydrogen (H_2_) and constitutes one of the most important sources of H_2_ on Earth (Reaction 1) (Truche et al., 2020).


(1) $$\begin{array}{l} \;{\left(\text{MgFe}\right)}_{2}{\text{SiO}}_{4}+H_{2}O\to {\text{Mg}}_{3}{\text{Si}}_{2}O_{5}{\left(\text{OH}\right)}_{4}+\text{Mg}{\left(\text{OH}\right)}_{2}+{\text{Fe}}_{3}O_{4}+H_{2}\\ \text{ Olivine Serpentinite Brucite Magnetite} \end{array}$$

The oxidation of ferrous iron in olivine or pyroxene to ferric iron and magnetite by water creates reducing conditions. Catalyzed by minerals, this facilitates abiotic reactions of the produced H_2_ with mantle-derived carbon dioxide (CO_2_) or carbon monoxide (CO) (McCollom and Seewald, 2001). In Sabatier (Reaction 2) and Fischer-Tropsch (Reaction 3) type processes, methane (CH_4_) and small organic molecules [C*_n_* H(2*n* + 2)] are enriched in the hydrothermal fluid (Barbier et al., 2020):


(2) $$4H_{2}+{\text{CO}}_{2}\to {\text{CH}}_{4}+2H_{2}O$$

(3) $$\left(3n+1\right)H_{2}+n{\text{CO}}_{2}\to C_{n}H_{2n+2}+2nH_{2}O$$

**Figure 1:**
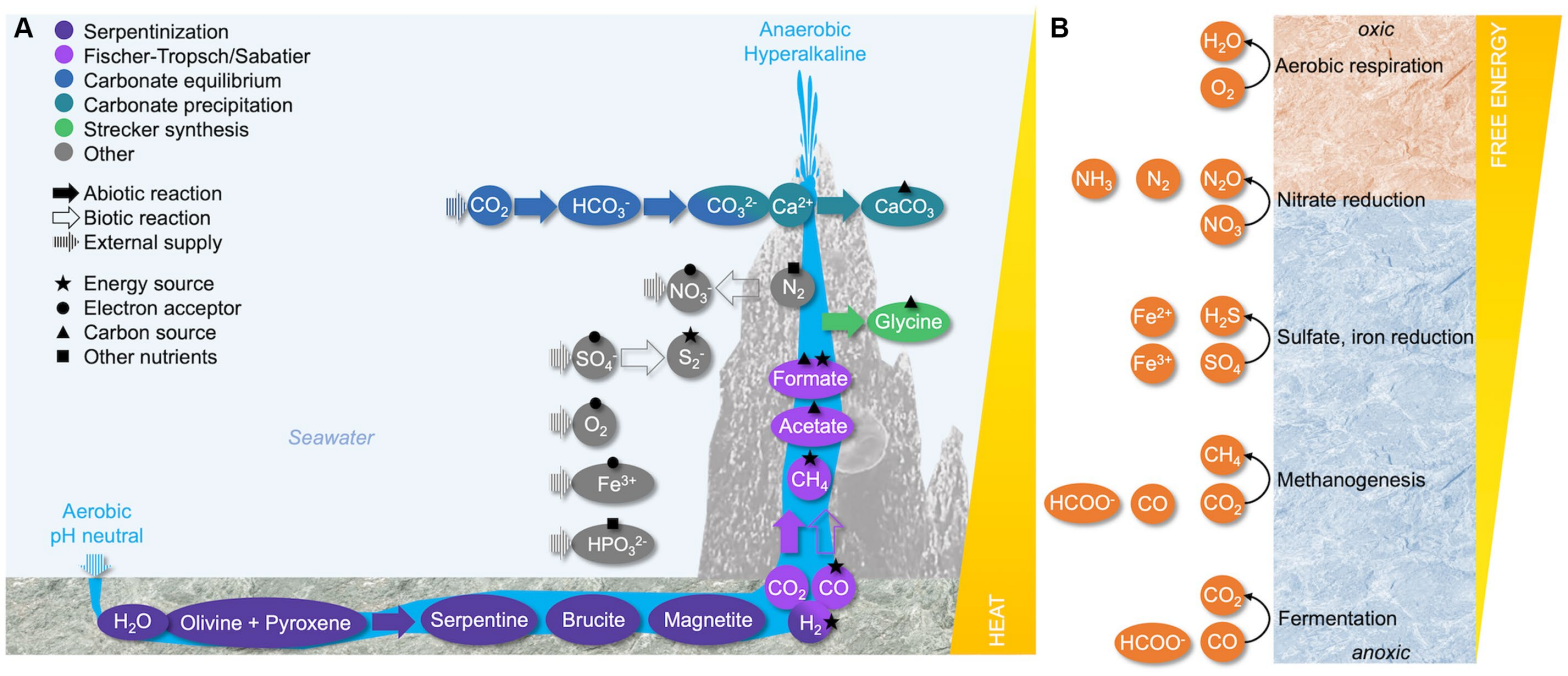
The biogeochemical environment of marine serpentinite-hosted systems. **(A)** Production of compounds of interest in microbial metabolism. Reactions are color-coded with solid arrows showing abiotic processes, empty arrows showing biotic processes, and striped arrows indicating external supply. The metabolic potential of shown compounds as an energy source, electron acceptor, carbon source, or other nutrient source is indicated with different shapes. Note that compound concentrations, external supply, temperature, and pH are strongly site dependent. In continental serpentinizing systems, travertines form instead of chimneys. **(B)** Redox potential of available electron acceptors along a gradient from the oxic surface to the anoxic interior of the chimney wall. Adapted from Boyd et al. (2014).

The serpentinization reaction produces hydrothermal fluids with pH values commonly surpassing 12. In these ultrabasic conditions, the carbonate equilibrium is permanently shifted from CO_2_ to carbonate species, removing most dissolved inorganic carbon (DIC) from the environment. Upon reaching the rock surface, much of the carbonate precipitates with fluid-derived calcium. Over time, calcium carbonate amalgamates with brucite and forms the chimneys or travertines typical for serpentinizing environments (Barnes et al., 1978; Früh-Green et al., 2004; McCollom and Seewald, 2013) (Figure 1A).

## 2. Serpentinite-hosted ecosystems

The products of serpentinization can support chemosynthetic microbial ecosystems growing independently from sunlight. Such serpentinite-hosted ecosystems are found in marine and continental environments, with hydrothermal fluids originating from marine, meteoric and/or groundwater sources.

Most marine serpentinizing ecosystems are located along rather slow-spreading mid-ocean ridges, where continuous tectonic activity facilitates frequent exposure of ultramafic rock (Schrenk et al., 2013; Albers et al., 2021). The most prominent example is the Lost City hydrothermal field near the Mid-Atlantic ridge (e.g., Kelley et al., 2005). A similar system, the Old City hydrothermal field, has recently been discovered along the Southwest Indian ridge (Lecoeuvre et al., 2021). Several other marine sites host mixed-type ecosystems that feature characteristics of both alkaline and acidic hydrothermal vents, such as the Rainbow (Flores et al., 2011), Logatchev (Perner et al., 2007), Ashadze (Fabri et al., 2011) and Kairei fields (Nakamura et al., 2009).

The most well-studied continental systems include the Samail ophiolite in Oman (Rempfert et al., 2017; Kraus et al., 2021), the Tablelands (Bay of Islands) ophiolite in Newfoundland (Brazelton et al., 2013), the Cabeço de Vide aquifer in Portugal (Tiago and Veríssimo, 2013), the Leka ophiolite complex in Norway (Daae et al., 2013), the Italian Gruppo di Voltri (Quéméneur et al., 2015), the Zambales ophiolite in the Philippines (Woycheese et al., 2015), The Cedars (Suzuki et al., 2013; Kohl et al., 2016) and Coast Range (Twing et al., 2017) ophiolites on the West Coast of the United States, the Chimaera (Tekirova) ophiolite in Turkey (Neubeck et al., 2017), the tropical Santa Elena ophiolite in Costa Rica (Crespo-Medina et al., 2017), the Del Puerto ophiolite in California (Blank et al., 2009) and the Troodos ophiolite in Cyprus (Rizoulis et al., 2016).

Terrestrial and marine serpentinizing systems are likely to differ in environmental variables such as salinity and the compounds available for microbial metabolism, which might influence the ecosystem’s overall functioning. An exciting transition site between terrestrial and marine serpentinite-hosted ecosystems is the Prony Bay Hydrothermal field located on the Southern Coast of New Caledonia, South Pacific (Launay and Fontes, 1985; Monnin et al., 2014). Prony Bay features several venting sites along a gradient from land to sea with a maximum depth of 50 m. The Prony Bay springs are fed by meteoric water, implying a strong salinity gradient between the hydrothermal fluid and ambient seawater (Monnin et al., 2014; Postec et al., 2015). Inversely, the Ney Springs system in Northern California features marine-type hydrothermal fluids in a continental context (Trutschel et al., 2022). Prony Bay is geochemically and microbiologically reflective of both ophiolitic and deep marine sites (Quéméneur et al., 2014; Postec et al., 2015; Frouin et al., 2018, 2022; Trutschel et al., 2022).

## 3. Metabolic strategies of serpentinite-hosted ecosystems

Serpentinite-hosted ecosystems are subjected to very challenging environmental conditions. The elevated pH poses a fundamental energetic problem on the cellular level, as it inverts the transmembrane pH gradient that typically drives all cellular processes. Furthermore, the high pH also reduces the bioavailability of electron acceptors, carbon, and other macronutrients (McCollom and Seewald, 2013; Schrenk et al., 2013). The microbial adaptations to these challenges are essential to understand life in a serpentinization context.

### 3.1. The challenge of maintaining bioenergetics

#### 3.1.1. Maintaining pH homeostasis and a proton motive force

The central challenge for life in hyperalkaline conditions revolves around maintaining intracellular pH homeostasis and, more critically, conserving an electrochemical proton gradient across the cell membrane, which is the main driving force of the cell. The so-called proton motive force has two components: A transmembrane pH gradient (ΔpH), which is usually alkaline inside the cell relative to the outside, and a transmembrane electrical potential (Δψ), which is negative as long as the inner membrane surface is negatively charged. Maintaining a circum-neutral intracellular pH is crucial to ensure the stability of nucleic acids and proteins. In hyperalkaline conditions, however, the ΔpH is inverted and very low due to H^+^ limitation outside of the cell, as the concentration of H^+^ decreases by 10^−4^ at pH 11 compared to pH 7. This reduces the proton motive force and jeopardizes pH homeostasis (Krulwich et al., 2011 and references therein). Most alkaliphiles employ mechanisms increasing the efficiency of H^+^ uptake while maintaining a high Δψ, which is also essential for pH homeostasis. This is achieved via K^+^/H^+^ and Na^+^/H^+^ antiporters with high H^+^ affinity, such as the Mrp complex in alkaliphilic *Bacillus* spp. (Ito et al., 2017). Those antiporters contribute to creating a transmembrane Na^+^ gradient, generating a sodium motive force that requires specialized sodium-F_1_F_0_-ATP synthases or Na^+^-dependent respiratory complexes (Krulwich et al., 2011; Kuhns et al., 2020). Based on varying Na^+^ concentrations, it is likely that these mechanisms differ between terrestrial and marine serpentinization-influenced site microorganisms. In low salt environments, alkaliphiles might excrete Na^+^ via V_1_V_0_-ATPases to maintain a sodium motive force (Suzuki et al., 2014; Ohlsson et al., 2019). Other alkaliphiles such as *Serpentinimonas* spp. isolated from The Cedars maintain a proton motive force using specialized H^+^ binding F type ATPases (Hicks et al., 2010; Suzuki et al., 2014).

#### 3.1.2. Energy conservation

The membrane potential generated via H^+^ or Na^+^ translocation depends on the redox potential of electron donors and acceptors. Serpentinization yields a range of reduced compounds that can serve as metabolic energy sources, most importantly H_2_ and CH_4_ (Figure 1A) (Boyd et al., 2014). The relative concentration of these gases varies significantly between sites (Etiope et al., 2011; Monnin et al., 2014), rendering generalized statements on a primary source of electrons provided by serpentinization difficult. While methanotrophs are more easily detected than hydrogenotrophs in many serpentinite-hosted environments (e.g., Brazelton et al., 2006; Kraus et al., 2021) (Table 1), the energetic potential of H_2_ oxidation greatly surpasses the oxidation potential of CH_4_. The detection of hydrogenotrophs may be limited by the methodological approach, as the metabolic potential to oxidize H_2_ cannot be predicted from 16S rRNA sequences (Brazelton et al., 2012, 2022). Accordingly, metagenomic surveys show that many organisms in all types of serpentinite-hosted systems feature [FeFe]- and [NiFe]-hydrogenases (Brazelton et al., 2012; Mei et al., 2016; Kraus et al., 2021; Lecoeuvre et al., 2021; Frouin et al., 2022) (Table 1).

**Table 1:** Taxonomic survey for critical metabolic functions in all types of serpentinite-hosted environments, specifying the methodological approach including metagenome-assembled genomes (MAGs) and single-cell amplified genomes (SAGs).

Metabolism	Taxon	Site	Method	References
Hydrogen oxidation	Bacteria_Pseudomonadota_Alphaproteobacteria^1^	The Cedars (shallow waters)	MAGs	Suzuki et al. (2017)
	Bacteria_Pseudomonadota_Gammaproteobacteria	The Cedars (shallow waters)	MAGs; 16S rRNA	Suzuki et al. (2013, 2017)
	Bacteria_Pseudomonadota_Gammaproteobacteria_Betaproteobacteriales (formerly Betaproteobacteria)^2^	The Cedars (shallow waters)	MAGs	Suzuki et al. (2017)
	Bacteria_Pseudomonadota_Gammaproteobacteria_Burkholderiales_Burkholderiaceae_Hydrogenophaga^3^	The Tablelands, Lost City (oxic/anoxic interface); Voltri; The Cedars (shallow waters); Prony Bay; Zambales	MAGs; 16S rRNA	Brazelton et al. (2012, 2017), Suzuki et al. (2013), Frouin et al. (2018), Woycheese et al. (2015)
	Bacteria_Pseudomonadota_Gammaproteobacteria_Burkholderiales_Burkholderiaceae_Serpentinimonas^4^	Voltri; The Cedars (shallow waters/non-specified)	16S rRNA; cultivation	Quéméneur et al. (2015), Suzuki et al. (2014, 2017), Brazelton et al. (2017), Bird et al. (2021)
	Bacteria_Pseudomonadota_Gammaproteobacteria_Burkholderiales_Burkholderiaceae_Serpentinimonas_S. raichei^5^	The Cedars	Cultivation	Bird et al. (2021)
	Bacteria_Pseudomonadota_Gammaproteobacteria_Burkholderiales_Burkholderiaceae_Serpentinimonas_S. barnesii^6^	The Cedars	Cultivation	Bird et al. (2021)
	Bacteria_Pseudomonadota_Gammaproteobacteria_Burkholderiales_Burkholderiaceae_Serpentinimonas_S. maccroryi^7^	The Cedars	Cultivation	Bird et al. (2021)
	Bacteria_Pseudomonadota_Gammaproteobacteria_Burkholderiales_Burkholderiaceae_Cupriavidus_C. necator (formerly *Ralstonia eutropha*)^8^	The Tablelands, Lost City (oxic/anoxic interface)	MAGs	Brazelton et al. (2012)
	Bacteria_Former class Deltaproteobacteria	The Cedars (shallow waters)	MAGs; 16S rRNA	Suzuki et al. (2013, 2017)
	Bacteria_Desulfobacterota_Desulfovibrionia_Desulfovibrionales^9^	Voltri (deep subsurface)	MAGs	Brazelton et al. (2017)
	Bacteria_Desulfobacterota_Desulfovibrionia_Desulfovibrionales_Desulfonatronaceae_Desulfonatronum^10^	Prony Bay	16S rRNA	Postec et al. (2015), Mei et al. (2016)
	Bacteria_BacillotaB_Desulfotomaculia_Desulfotomaculales_ Desulfotomaculaceae_Desulfotomaculum_D. alkaliphilum^11^	Lost City	16S rRNA	Brazelton et al. (2006, 2010)
	Archaea_Methanobacteriota_Methanobacteria_Methanobacteriales_Methanobacteriaceae_Methanobacterium_Lineage type I	Voltri; Samail (surface waters); Prony Bay	MAGs, SAGs, 14C labelling	Quéméneur et al. (2015, 2021, 2023), Fones et al. (2021)
Aerobic methane oxidation	Archaea_Methanobacteriota_Methanobacteria_Methanobacteriales_Methanobacteriaceae_Methanobacterium_M. alkalithermotolerans strain DSM102889	La Crouen	Cultivation	Mei et al. (2022)
	Bacteria_Pseudomonadota_Gammaproteobacteria_Methylococcales_Methylococcaceae^12^	Voltri (shallow subsurface mixing zone)	16S rRNA, MAGs, 13C labelling	Brazelton et al. (2017)
	Bacteria_Pseudomonadota_Gammaproteobacteria_Methylococcales_Methylococcaceae_Methylococcus	Samail	16S rRNA	Kraus et al. (2021)
	Bacteria_Pseudomonadota_Alphaproteobacteria_Rhizobiales_Beijerinckiaceae_Methylosinus	Voltri	16S rRNA	Quéméneur et al. (2015)
Methanogenesis/anaerobic methane oxidation	Archaea_Halobacteriota_Methanomicrobia_Methanomicrobiales	Santa Elena	16S rRNA (MAGs)	Crespo-Medina et al. (2017)
	Archaea_Methanobacteriota_Methanobacteria_Methanobacteriales	Santa Elena; Voltri	16S rRNA (MAGs)	Crespo-Medina et al. (2017), Quéméneur et al. (2015)
	Archaea_Methanobacteriota_Methanobacteria_Methanobacteriales_Methanobacteriaceae^13^	Voltri (deep subsurface)	16S rRNA, MAGs, 13C labelling	Brazelton et al. (2017)
	Archaea_Methanobacteriota_Methanobacteria_Methanobacteriales_Methanobacteriaceae_Methanobacterium	Samail (subsurface); Voltri; Zambales	16S rRNA; MAGs; SAGs; 13C labelling; 14C labelling	Kraus et al. (2021), Brazelton et al. (2017), Fones et al. (2021), Woycheese et al. (2015), Quéméneur et al. (2015)
	Archaea_Methanobacteriota_Methanobacteria_Methanobacteriales_Methanobacteriaceae_Methanobacterium_M. alcaliphilum strain DSM3387	Del Puerto	16S rRNA	Blank et al. (2009)
	Archaea_Methanobacteriota_Methanobacteria_Methanobacteriales_Methanobacteriaceae_Methanobacterium_M. alkalithermotolerans strain DSM102889	La Crouen	Cultivation	Mei et al. (2022)
	Archaea_Halobacteriota_Methanosarcinia_Methanosarcinales	Santa Elena; Voltri	16S rRNA (MAGs)	Crespo-Medina et al. (2017), Quéméneur et al. (2015), Suzuki et al. (2013)
	Archaea_Halobacteriota_Methanosarcinia_Methanosarcinales_LCMS phylotype^14^	Lost City (Brazelton et al. (2006): High-temperature); Prony Bay (intertidal and submarine); Old City	16S rRNA, MAGs; 13C labelling	Schrenk et al. (2004), Brazelton et al. (2006, 2011), Frouin et al. (2018), Lecoeuvre et al. (2021)
	Archaea_Halobacteriota_Methanosarcinia_Methanosarcinales_TCMS phylotype	The Cedars; Prony Bay (intertidal and submarine); Old City	16S rRNA, MAGs	Suzuki et al. (2013), Frouin et al. (2018), Lecoeuvre et al. (2021)
	Archaea_Halobacteriota_Syntropharchaeia_ANME-1	Lost City (low temperature); Santa Elena; Cabeço de Vide	16S rRNA (MAGs)	Brazelton et al. (2006), Crespo-Medina et al. (2017), Tiago and Veríssimo (2013)
Formate consumption	Bacteria_Pseudomonadota_Gammaproteobacteria_Methylococcales_Methylococcaceae^12^	Voltri (shallow subsurface mixing zone)	MAGs	Brazelton et al. (2017)
	Bacteria_Desulfobacterota_Desulfovibrionia_Desulfovibrionales^9^	Voltri (deep subsurface)	MAGs	Brazelton et al. (2017)
	Bacteria_Ca. Lithacetigena^15^	The Cedars, Hakuda Happo hot springs	MAGs	Nobu et al. (2022)
	Bacteria_Ca. Bipolaricaulota (OP1/MSBL6)^17^	Lost City	MAGs	Brazelton et al. (2022)
	Archaea_Methanobacteriota_Methanobacteria_Methanobacteriales_Methanobacteriaceae_Methanobacterium_Lineage type II	Samail (subsurface)	MAGs, SAGs, 14C labelling	Fones et al. (2021)
Acetate consumption	Bacteria_Desulfobacterota_Desulfovibrionia_Desulfovibrionales^9^	Voltri (deep subsurface)	MAGs	Brazelton et al. (2017)
	Archaea_Halobacteriota_Methanosarcinia_Methanosarcinales_LCMS phylotype^14^	Lost City	MAGs	Brazelton et al. (2011)
Glycine consumption	Bacteria_Ca. Lithacetigena^15^	The Cedars, Hakuda Happo hot springs	MAGs	Nobu et al. (2022)
Calcium carbonate consumption	Bacteria_Pseudomonadota_Gammaproteobacteria_Burkholderiales_Burkholderiaceae_Serpentinimonas^4^	The Cedars	Cultivation	Suzuki et al. (2014)
	Bacteria_Pseudomonadota_Gammaproteobacteria_Burkholderiales_Burkholderiaceae_Serpentinimonas_S. raichei^5^	The Cedars	Cultivation	Bird et al. (2021)
	Bacteria_Pseudomonadota_Gammaproteobacteria_Burkholderiales_Burkholderiaceae_Serpentinimonas_S. barnesii^6^	The Cedars	Cultivation	Bird et al. (2021)
	Bacteria_Pseudomonadota_Gammaproteobacteria_Burkholderiales_Burkholderiaceae_Serpentinimonas_S. maccroryi^7^	The Cedars	Cultivation	Bird et al. (2021)
	Bacteria_ NPL-UPA2 clade^18^	The Cedars, Prony Bay, Lost City	MAGs	Suzuki et al. (2018), Brazelton et al. (2022)
	Bacteria_Ca. Bipolaricaulota (OP1/MSBL6)^17^	Lost City	MAGs	Brazelton et al. (2022)
CO oxidation	Bacteria_Pseudomonadota_Gammaproteobacteria_Burkholderiales_Burkholderiaceae_Hydrogenophaga^3^	The Tablelands, Lost City (oxic/anoxic interface)	MAGs; 16S rRNA, 13C labelling	Brazelton et al. (2012), Morrill et al. (2014)
	Bacteria_Pseudomonadota_Gammaproteobacteria_Burkholderiales_Burkholderiaceae_Cupriavidus_C. necator (formerly *Ralstonia eutropha*)^8^	The Tablelands, Lost City (oxic/anoxic interface)	MAGs	Brazelton et al. (2012)
	Bacteria_Actinobacteriota_Ca. Hakubanella thermoalkaliphilus	Hakuda Happo hot springs	16S rRNA, SAGs	Merino et al. (2020)
	Archaea_Hadesarchaea (formerly SAGMEG)	Prony Bay	16S rRNA	Postec et al. (2015)
Sulfide/sulfur oxidation	Bacteria_Pseudomonadota_Gammaproteobacteria_Betaproteobacteriales (formerly Betaproteobacteria)^2^	Cabeço de Vide	16sS rRNA (DGGE)	Tiago and Veríssimo (2013)
	Bacteria_Pseudomonadota_Gammaproteobacteria_Thiomicrospirales_Thiomicrospiraceae_Hydrogenovibrio (formerly Thiomicrospira)	Lost City (low temperature)	16S rRNA	Brazelton et al. (2006)
	Bacteria_Pseudomonadota_Alphaproteobacteria_Rhodobacterales_Rhodobacteraceae	Ney Springs	MAGs, Cultivation	Trutschel et al. (2022)
	Bacteria_Pseudomonadota_Gammaproteobacteria_Pseudomonadales_Halomonadaceae_Halomonas	Ney Springs	MAGs, Cultivation	Trutschel et al. (2022)
Sulfate reduction	Bacteria_BacillotaA_Clostridia^16^	Cabeço de Vide; The Cedars (deep subsurface)	16S rRNA (DGGE)	Tiago and Veríssimo (2013), Suzuki et al. (2013)
	Bacteria_BacillotaB_Desulfotomaculia_Desulfotomaculales_ Desulfotomaculaceae_Desulfotomaculum_D. alkaliphilum^11^	Lost City	16S rRNA	Brazelton et al. (2006, 2010)
	Bacteria_BacillotaD_Dethiobacteria_Dethiobacterales_Dethiobacteraceae_Dethiobacter	The Tablelands, The Cedars, Cabeço de Vide, Prony Bay, Zambales		Brazelton et al. (2012), Suzuki et al. (2013), Tiago and Veríssimo (2013), Postec et al. (2015), Woycheese et al. (2015), Crespo-Medina et al. (2017), Trutschel et al. (2022), Twing et al. (2017)
	Bacteria_Desulfobacterota_Desulfovibrionia_Desulfovibrionales^9^	Voltri	MAGs	Brazelton et al. (2017)
	Bacteria_Desulfobacterota_Desulfovibrionia_Desulfovibrionales_Desulfonatronaceae_Desulfonatronum^10^	Prony Bay	16S rRNA	Postec et al. (2015), Mei et al. (2016)
	Bacteria_Nitrospirota_Thermodesulfovibrionia_Thermodesulfovibrionales_Thermodesulfovibrionaceae	Samail (subsurface fluids)	16S rRNA	Rempfert et al. (2017)
Nitrogen fixation	Bacteria_Pseudomonadota_Alphaproteobacteria_Azospirillales_Azospirillaceae_Azospirillum	Voltri	16S rRNA	Quéméneur et al. (2015)
	Archaea_Methanobacteriota_Methanobacteria_Methanobacteriales_Methanobacteriaceae^13^	Voltri (deep subsurface)	16S rRNA, MAGs	Brazelton et al. (2017)
	Archaea_Halobacteriota_Methanosarcinia_Methanosarcinales_LCMS phylotype^14^	Lost City	MAGs	Brazelton et al. (2011)
Phosphonate catabolism	Bacteria_Proteobacteria_Alphaproteobacteria^1^	Prony Bay, Lost City	MAGs	Frouin et al. (2022)
	Bacteria_Proteobacteria_Gammaproteobacteria_Betaproteobacteriales (formerly Betaproteobacteria)^2^	Coast Range, Voltri, Santa Elena, Cabeço de Vide	MAGs	Frouin et al. (2022)
	Bacteria_ BacillotaA _Clostridia^16^	Prony Bay, Lost City	MAGs	Frouin et al. (2022)
Acetogenesis	Bacteria_Chloroflexota (formerly Chloroflexi)	The Cedars (deep subsurface); Prony Bay (submarine)	MAGs; 16S rRNA	Suzuki et al. (2013, 2017), Frouin et al. (2018)
	Bacteria_ NPL-UPA2 clade^18^	The Cedars; Prony Bay	16S rRNA; MAGs	Postec et al. (2015), Mei et al. (2016), Suzuki et al. (2018)
	Bacteria_BacillotaD_Dethiobacteria_Dethiobacterales_Dethiobacteraceae_Dethiobacter_D. alkaliphilus	The Cedars; Prony Bay; Cabeço de Vide	16S rRNA	Postec et al. (2015), Mei et al. (2016), Suzuki et al. (2013), Tiago and Veríssimo (2013)
	Bacteria_Ca. Bipolaricaulota (OP1/MSBL6)^17^	Samail (subsurface fluids)	MAGs	Colman et al. (2022)
Fermentation	Bacteria_ BacillotaA _Clostridia	The Tablelands, Lost City	MAGs	Brazelton et al. (2012)
	Bacteria_ BacillotaA _Clostridia_Thermoanaerobacterales_Candidate Division OD1	The Cedars (deep subsurface); Voltri	16S rRNA, MAGs, 13C labelling	Suzuki et al. (2013, 2017), Brazelton et al. (2017)
	Bacteria_ BacillotaA _Clostridia_Lachnospirales_Vallitaleaceae_Vallitalea_V. pronyensis	Prony Bay	Cultivation	Ben Aissa et al. (2014), Mei et al. (2014)
	Bacteria_ BacillotaA _Clostridia_Peptostreptococcales_Natronincolaceae_Alkaliphilus_A. hydrothermalis	Prony Bay	Cultivation	Ben Aissa et al. (2015)
	Bacteria_ BacillotaA _Clostridia_Peptostreptococcales_Natronincolaceae_Alkaliphilus_A. serpentinus	Prony Bay	Cultivation	Postec et al. (2021)
	Bacteria_ BacillotaA _Clostridia_Peptostreptococcales_Natronincolaceae_Alkaliphilus_A. pronyensis	Prony Bay	Cultivation	Postec et al. (2021)
	Bacteria_ BacillotaA _Clostridia_Peptostreptococcales_Natronincolaceae_Serpentinicella_S. alkaliphila	Prony Bay	Cultivation	Mei et al. (2016)
	Bacteria_ BacillotaA _Clostridia_Peptostreptococcales_Peptostreptococcaceae_Acetoanaerobium_A. pronyense	Prony Bay	Cultivation	Bes et al. (2015)
	Bacteria_Bacteroidetes_Bacteroidia_Bacteroidales_ML635J-40	Voltri	16S rRNA, MAGs, 13C labelling	Brazelton et al. (2017)
	Bacteria_BacillotaA_Clostridia_Peptostreptococcales_Peptostreptococcaceae	Ney Springs	MAGs, 16S rRNA	Trutschel et al. (2022)
	Bacteria_BacillotaA_Clostridia_Peptostreptococcales_Tindalliaceae_Tindallia	Ney Springs	MAGs, 16S rRNA	Trutschel et al. (2022)
	Bacteria_BacillotaB_Desulfotomaculia_Desulfotomaculales_ Desulfotomaculaceae_Desulfotomaculum	The Tablelands, Lost City	MAGs	Brazelton et al. (2012)
	Bacteria_Bacilli_Bacillota_Erysipelotrichales_Erysipelotrichaceae	The Tablelands	MAGs	Brazelton et al. (2012)
Iron reduction	Bacteria_ BacillotaA _Clostridia_Peptostreptococcales_Natronincolaceae_Alkaliphilus	Troodos	Cultivation	Rizoulis et al. (2016)
	Bacteria_Bacillota_Bacilli_Paenibacillales_Paenibacillaceae_Paenibacillus	The Cedars	Cultivation	Rowe et al. (2017)
Photosynthesis	Bacteria_Cyanobacteria_Cyanobacteriia_Leptolyngbyales_Leptolyngbyaceae_Leptolyngbya	Voltri (surface); Del Puerto	16S rRNA	Kamran et al. (2020), Blank et al. (2009)
	Bacteria_Cyanobacteria_Cyanobacteriia_Synechococcales_Synechococcaceae_Synechococcus	Prony Bay	16S rRNA	Mei et al. (2016)

Taxa appearing in several metabolic groups on the same taxonomic level are assigned with superscript numbers. The taxonomy is based on the Genome Taxonomy Database (GTDB Release 214).

Another potential electron source in serpentinizing systems is constituted by compounds not directly created by serpentinization such as reduced sulfur species (Sabuda et al., 2020; Trutschel et al., 2022) and CO (Brazelton et al., 2012; Morrill et al., 2014; Fones et al., 2019) (Table 1). While the oxidation potential of CO is very low, the ability to use this energy source may provide a valuable ecological advantage. In surface exposed serpentinization-influenced waters, light constitutes an additional energy source used by cyanobacterial phototrophs (e.g., Kamran et al., 2020) (Table 1).

While serpentinization provides an abundance of electron donors, the availability of terminal electron acceptors is limited, especially in terrestrial serpentinizing systems, and mainly derived from the ambient environment (Figure 1A). Oxygen represents a very potent electron acceptor on the chimney or travertine surface. With increasing proximity to the reduced hydrothermal endmember, however, the availability of oxygen or alternative electron acceptors sharply decreases. The microbial community near the oxic-anoxic interphase may use nitrate (Frouin et al., 2022), even though data on nitrate reduction is scarce. In addition, organisms from the Troodos and The Cedars ophiolites have been shown to reduce metals such as iron or magnetite (Rizoulis et al., 2016; Rowe et al., 2017) (Table 1). Towards the anoxic interior of the hydrothermal carbonate chimneys or in deep ophiolite groundwaters, sulfate reduction is a dominant metabolic strategy in all types of serpentinite-hosted systems (Brazelton et al., 2006; Tiago and Veríssimo, 2013; Postec et al., 2015; Glombitza et al., 2021) (Table 1 and Figure 1B).

To deal with electron acceptor limitation, many microbes also perform fermentation of sugars, simple organic acids and amino acids, including Stickland type reactions (Barker, 1981; Postec et al., 2021). Therefore, a large proportion of the anaerobic serpentinite-hosted community may not feature a *bona fide* electron transport chain with cytochromes or quinones (Table 1). Genomic analysis suggests that many serpentinite-hosted fermenters conserve energy by substrate-level phosphorylation (e.g., in glycolysis), or via bifurcative-confurcative [FeFe] H_2_-producing hydrogenases, which balance the reducing equivalents NADH and ferredoxin produced by fermentation (Westphal et al., 2018). This is often associated with the Rnf complex, a respiratory enzyme that catalyzes the oxidation of reduced ferredoxin to the reduction of NAD^+^. The negative free energy change of this reaction is used to generate a transmembrane H^+^ or Na^+^ gradient (Westphal et al., 2018). This system can be considered a primitive respiratory mechanism where the terminal electron acceptor is H^+^ (Buckel and Thauer, 2018).

### 3.2. The challenge of coping with nutrient limitation

Next to maintaining energy-yielding reactions, microorganisms in serpentinite-hosted environments must cope with severe nutrient limitation resulting from the decreased solubility of essential macronutrients at high pH (McCollom and Seewald, 2013; Schrenk et al., 2013). Especially relevant for the metabolic functioning of the community are the carbon, nitrogen, and phosphorous sources for primary production (Figure 1A).

#### 3.2.1. Carbon sources and carbon fixation

One of the most significant issues regarding primary production in serpentinite-hosted environments is the absence of DIC, which precipitates as calcium carbonate in hyperalkaline conditions. While calcium carbonate is mostly insoluble and thus unavailable as a carbon source, it has nevertheless been shown to support the growth of some serpentinite-hosted microorganisms. This might be the result of local redissolution into bicarbonate catalyzed by the carbonic anhydrase (Suzuki et al., 2014; Fones et al., 2019; Bird et al., 2021) (Table 1). Alternatively, small organic molecules may serve as primary source of carbon. These include organic acids such as formate and acetate produced in Fischer-Tropsch and Sabatier-type reactions (Barbier et al., 2020; Fones et al., 2021) or via acetogenesis and fermentation (Kohl et al., 2016; Suzuki et al., 2017), as well as amino acids such as glycine produced in Strecker synthesis (Ménez et al., 2018; Nobu et al., 2022) (Figure 1A). The abiotic origin of those organic carbon sources tackles the definition of heterotrophy, which normally refers to the consumption of organic compounds derived from organic sources (Schönheit et al., 2016).

While bicarbonate, formate, acetate, and glycine have been shown to support the growth of microorganisms associated with serpentinization, their metabolic route remains hypothetical. Transferred across the cell membrane via specialized transporters, formate can be oxidized to CO_2_ via the formate dehydrogenase in the pH-neutral cytoplasm (Brazelton et al., 2022). Likewise, bicarbonate can be reduced to CO_2_ via the carbonic anhydrase (Suzuki et al., 2014; Bird et al., 2021). The produced CO_2_ is subsequently introduced to different carbon fixation pathways yielding acetyl-CoA. In serpentinite-hosted environments, the Wood-Ljungdahl pathway, reverse tricarboxylic acid cycle, and Calvin-Benson-Bassham cycle have been confirmed (Seyler et al., 2020). Based on a recent study expanding the phylogenetic range of most carbon fixation pathways, the 3-hydroxypropionate bi-cycle, dicarboxylate/4-hydroxybutyrate cycle, and 3-hydroxypropionate/4-hydroxybutyrate cycle might also be employed (Garritano et al., 2022). Contrary to formate and bicarbonate, glycine can be directly transformed into acetyl-phosphate and subsequently acetyl-coA via the lesser known reductive glycine pathway (Sánchez-Andrea et al., 2020). Genes encoding the glycine reductase are found in metagenomes from Lost City, The Cedars, and the Japanese Hakuba Happo hot springs (Brazelton et al., 2022; Nobu et al., 2022). Also acetate can be directly transformed into acetyl-phosphate and acetyl-CoA, rendering its metabolic route less complex (Rose et al., 1954).

#### 3.2.2. Sources of other nutrients

Serpentinization also decreases the solubility of other macronutrients essential for microbial growth. Inorganic phosphorous is severely limited in serpentinizing environments because it is scavenged by the mineral brucite (Schrenk et al., 2013). A metagenomic survey revealed the high occurrence of genes involved in phosphonate catabolism in serpentinizing sites, suggesting that the microbial community might use phosphonates as an alternative phosphorous source (Frouin et al., 2022). The catabolism of methylphosphonate, the most commonly available phosphonate species in marine environments, may additionally contribute to the global carbon and energy budget in these ecosystems by releasing CH_4_ (Frouin et al., 2022). On the contrary, the availability of nitrogen in serpentinizing environments remains controversial. While some authors suggest that concentrations are low (Schrenk et al., 2013), others propose that N_2_ and nitrate are readily available to the serpentinite-hosted community (Lang et al., 2013; Rempfert et al., 2023). Potential nitrogen limitation may be alleviated by the fixation of N_2_ derived from the endmember fluids or ambient seawater (Morrill et al., 2013; Monnin et al., 2014) (Figure 1A). While a recent study found the associated genetic marker *nifH* in 10 different serpentinite-hosted systems, its overall abundance was low (Frouin et al., 2022).

### 3.3. Metabolic links to the emergence of life

The biochemical characteristics of serpentinite-hosted ecosystems reinforce the presumed link between serpentinization and the beginnings of life. Serpentinization is an ancient process which likely occurred on early Earth (Russell et al., 2010). The abiotic production of organic acids associated with serpentinization is for instance supported by isotopic signatures (McCollom and Seewald, 2013). Likewise, amino acids such as glycine may be formed abiotically, which is especially interesting in prebiotic chemistry (Aubrey et al., 2009; Ménez et al., 2018). The metabolic use of these compounds is linked to very deep-branching functions, such as the reductive tricarboxylic acid cycle and the Wood-Ljungdahl pathway, which are likely the most ancient carbon fixation pathways on Earth (Sumi and Harada, 2021). Another primordial function preserved in serpentinizing environments is CO oxidation. CO is not only one of the most ancient energy sources exploited in metabolism but is also suggested to have played a key role in several critical prebiotic reactions (King and Weber, 2007). It may thus constitute a direct link between abiotic and biotic chemistry.

## 4. Diversity of identified metabolic key players

The specific metabolic challenges posed by serpentinization suggest the presence of specialized taxonomic groups playing an important role in the trophic network. It might be possible that such “core” taxa are relevant in a wide variety of serpentinizing environments, even though the overall community structure can vary significantly in space and time (Suzuki et al., 2013; Postec et al., 2015; Fones et al., 2019; Brazelton et al., 2022; Trutschel et al., 2022).

The community of H_2_ oxidizers appears to be dominated by Gammaproteobacteria (Table 1). This includes a major proportion of *Serpentinimonas* (formerly grouped under Betaproteobacteria). *Serpentinimonas* is one of the taxa most commonly associated with serpentinization, and represented by some of the few available isolates from serpentinite-hosted ecosystems (Suzuki et al., 2014; Bird et al., 2021). So far, all of those isolated strains originate from The Cedars. Still, 16S rRNA analysis confirms the presence of *Serpentinimonas* and its sister genus *Hydrogenophaga* in other terrestrial systems, as well as in Prony Bay and Lost City (e.g., Brazelton et al., 2012; Quéméneur et al., 2015; Woycheese et al., 2015; Frouin et al., 2018) (Table 1).

While aerobic methane oxidation is mostly performed by the bacterial *Methylococcales* (Brazelton et al., 2017; Kraus et al., 2021), anaerobic methanotrophy and methanogenesis feature exclusively archaea (Table 1). Hydrogenotrophic methanogens belonging to *Methanobacteriales* are often detected in serpentinite-hosted terrestrial ecosystems (Woycheese et al., 2015; Brazelton et al., 2017; Kraus et al., 2021; Quéméneur et al., 2021, 2023; Mei et al., 2022). In addition, there is a subgroup of *Methanosarcinales* which is probably endemic to serpentinizing environments and includes two distinct phylotypes (e.g., Schrenk et al., 2004; Brazelton et al., 2010; Suzuki et al., 2013). Next to their systems of origin, The Cedars *Methanosarcinales* (TCMS) and Lost City *Methanosarcinales* (LCMS) have been observed in Prony Bay (Frouin et al., 2018) and Old City (Lecoeuvre et al., 2021) (Table 1). However, attempts to culture them have been unsuccessful so far.

The fermenting community seems almost entirely dominated by *Clostridia* (Table 1), of which several novel species have been isolated from Prony Bay (Ben Aissa et al., 2014, 2015; Mei et al., 2014; Bes et al., 2015; Postec et al., 2021).

Other energy yielding metabolic strategies including CO oxidation, sulfur oxidation and sulfate reduction are performed by a broader diversity of taxonomic groups (Table 1). Notably, the community of sulfate reducers includes *Desulfovibrionales* and *Dethiobacter* species, which can be very abundant in serpentinizing environments (Brazelton et al., 2012; Suzuki et al., 2013; Tiago and Veríssimo, 2013; Postec et al., 2015; Woycheese et al., 2015; Mei et al., 2016) (Table 1).

Regarding carbon uptake, it might be especially interesting to further investigate certain candidate phyla that occur in various serpentinizing systems. For example, *Ca. Bipolaricaulota* can use bicarbonate and formate (Brazelton et al., 2022) and plays a role in acetogenesis (Colman et al., 2022). Likewise, *Ca.* NPL-UPA2 grows on bicarbonate (Brazelton et al., 2022) and performs acetogenesis via the Wood-Ljungdahl pathway (Suzuki et al., 2018) (Table 1). The Wood-Ljungdahl pathway is also employed by *Ca. Hakubanella thermoalkaliphilus*, a novel Actinobacteriota from the Hakuba Happo hot springs serpentinizing system (Merino et al., 2020). Finally, *Ca.* Lithacetigena was recently shown to perform glycine reduction (Nobu et al., 2022). These candidate phyla might play an important role in the trophic chain by supplying fixed carbon to the community.

## 5. Methodological shortcomings and future challenges

While several critical metabolic strategies and taxonomic groups could be identified, the scope of their distribution across serpentinizing ecosystems remains unclear due to methodological biases and shortcomings. Firstly, continental sites have been studied much more extensively than marine ones (Table 1). Our understanding of the latter is almost entirely based on the famous Lost City (Table 1) and most recently Old City (Lecoeuvre et al., 2021), which limits the generalization of findings on marine serpentinizing systems and reduces the meaningfulness of comparison with continental ones. This issue emphasizes the interest of the shallow marine transition field of Prony Bay. Its common characteristics with continental and marine sites may help establish the core metabolic properties of serpentinite-hosted ecosystems. In addition, the study of such shallow fields is facilitated by their geographical accessibility.

Another factor introducing bias is the methodological approach. Most studies rely on metabarcoding and metagenomic techniques (Table 1), which are strongly dependent on the scope of available reference databases. Moreover, the presence of a functional gene does not necessarily signify its activity. However, confirmation of gene expression is rare, notably due to technical difficulties in obtaining quality metatranscriptomes from such environments (Table 1). In addition, there is a lack of experimental evidence complementing bioinformatic hypotheses. Studies attempting to bridge this gap include activity measurements using ^13^C and ^14^C labeled substates in microcosms (Brazelton et al., 2011, 2017; Morrill et al., 2014; Fones et al., 2021), as well as isolation of *Serpentinimonas* and *Clostridia* species from cultures (e.g., Suzuki et al., 2014; Postec et al., 2021) (Table 1). Their small number is probably also associated with technical difficulties, including the cultivation of recalcitrant microorganisms such as obligate anaerobic chemolithoautotrophs. While combined omics approaches can provide valuable results, critical metabolic groups will eventually need to be cultivated to confirm their functional role in the ecosystem. This may be facilitated by implementing more sophisticated culture platforms to mimic the conditions associated with serpentinization in the laboratory.

## 6. Conclusion

Serpentinite-hosted environments are inhabited by microbial communities that cope with energetic challenges and severe nutrient limitation. It can be assumed that a significant proportion of those microorganisms yield energy from H_2_ oxidation with electron acceptors derived from external sources or CO_2_ degassed from mantle rocks. Calcium carbonate can serve as inorganic carbon source, and formate, acetate and glycine as organic carbon sources for primary production. Bicarbonate, formate, and glycine may be fixed via different carbon fixation pathways such as the Wood-Ljungdahl pathway, the reverse tricarboxylic acid and Calvin-Benson-Bassham cycles and the reductive glycine pathway. Moreover, the microbial communities might cope with nitrogen and phosphorous limitation by fixing N_2_ and breaking down phosphonates. The analysis of functional genes suggests that taxa such as *Gammaproteobacteria*, *Desulfovibrionales*, *Clostridia* and several candidate phyla play a crucial role in the trophic network and that the genera *Serpentinimonas*, *Hydrogenophaga*, and *Methanobacterium* as well as uncultivated *Methanosarcinales*, are characteristic for serpentinizing environments. However, the scope of research on serpentinite-hosted ecosystems needs to be broadened by including a greater diversity of marine and shallow transition sites. In addition, experimental evidence is needed to confirm the metabolic activity of hypothesized key players. A technological advancement of the methodological approach might not only contribute to the understanding of present serpentinite-hosted ecosystems, but also provide insights into the beginning of life on Earth and potentially elsewhere.

## Author contributions

GE, AP, and RP: conceptualization. AL and MQ: validation. RP: investigation and writing of original draft. AL, MQ, AP, GE, and RP: review and edit of original draft. GE and AP: supervision. GE: project administration. All authors contributed to the article and approved the submitted version.

## Funding

This project was financially supported by the ANR MICROPRONY (N°19-CE02-0020-02), the French Institute of Research for Development (IRD), and a Ph.D. fellowship granted to RP by the Aix-Marseille University’s Doctoral School “Sciences de l’Environnement” (ED 251).

## Conflict of interest

The authors declare that the research was conducted in the absence of any commercial or financial relationships that could be construed as a potential conflict of interest.

## Publisher’s note

All claims expressed in this article are solely those of the authors and do not necessarily represent those of their affiliated organizations, or those of the publisher, the editors and the reviewers. Any product that may be evaluated in this article, or claim that may be made by its manufacturer, is not guaranteed or endorsed by the publisher.
